# La vergüenza por la pérdida de dientes: entre el sufrimiento psíquico y la desigualdad estructural en la salud oral chilena

**DOI:** 10.18294/sc.2026.5911

**Published:** 2026-03-19

**Authors:** Manuel Alejandro Castro Gatica, Karen Danke Hausdorf, Ingeborg Bevensee Canavati, Ana Beatriz Silva Lopes, Jorge Gamonal

**Affiliations:** 1 Doctor en Sociología. Docente investigador, profesor asistente, Facultad de Ciencias Sociales, Departamento de Trabajador Social, Universidad Alberto Hurtado, Santiago, Chile. macastrog@uahurtado.cl Universidad Alberto Hurtado Facultad de Ciencias Sociales Departamento de Trabajador Social Universidad Alberto Hurtado Santiago Chile macastrog@uahurtado.cl; 2 Doctora en Ciencias Odontológicas. Docente investigadora, Facultad de Odontología, Universidad San Sebastián, Santiago, Chile. karen.danke@uss.cl Universidad San Sebastián Facultad de Odontología Universidad San Sebastián Santiago Chile karen.danke@uss.cl; 3 Odontóloga. Rehabilitadora oral. Investigadora adjunta, Universidad de Chile, Santiago, Chile. Ibevensee@odontologia.uchile.cl Universidad de Chile Universidad de Chile Santiago Chile Ibevensee@odontologia.uchile.cl; 4 Doctora en Ciencias Odontológicas. Profesora asistente, Facultad Odontología y Ciencias de la Rehabilitación, Universidad San Sebastián, Santiago, Chile. ana.silva@uss.cl Universidad San Sebastián Facultad Odontología y Ciencias de la Rehabilitación Universidad San Sebastián Santiago Chile ana.silva@uss.cl; 5 Doctor en Ciencias Odontológicas. Profesor titular, Facultad de Odontología, Centro de Epidemiología y Vigilancia de las Enfermedades Orales (CEVEO), Universidad de Chile, Santiago, Chile. jgamonal@odontologia.uchile.cl Universidad de Chile Facultad de Odontología Centro de Epidemiología y Vigilancia de las Enfermedades Orales (CEVEO) Universidad de Chile Santiago Chile jgamonal@odontologia.uchile.cl

**Keywords:** Salud Mental, Vergüenza, Salud Bucal, Diente, Chile, Mental Health, Shame, Oral Health, Tooth, Chile

## Abstract

El artículo analiza cómo la pérdida dental en adultos mayores no es solo un problema clínico, sino un fenómeno social y emocional marcado por la vergüenza, el estigma y la desigualdad estructural en la salud oral chilena. Desde un enfoque cualitativo de tipo narrativo, con un alcance descriptivo y una orientación hermenéutica (interpretativa), se realizaron 12 entrevistas en la comuna Cerro Navia, en la Región Metropolitana de Chile, entre agosto y septiembre de 2024. En los relatos se evidencia que la ausencia de dientes impacta en la autoestima, la identidad y la participación social. La vergüenza se manifiesta en conductas como cubrirse la boca, evitar sonreír, hablar en público o asistir a reuniones, lo que genera aislamiento social y sufrimiento psíquico. Los testimonios muestran que la falta de recursos económicos y el limitado acceso a atención dental pública profundizan este malestar, obligando a muchas personas a resignarse a la pérdida dental. Esta condición, además de limitar la alimentación y la funcionalidad cotidiana, se convierte en un marcador social de clase, reforzando juicios morales asociados a la pobreza y la falta de higiene. La investigación concluye que la vergüenza no puede comprenderse únicamente como una emoción individual, sino como un dispositivo social que reproduce desigualdades y exclusiones. Se propone un enfoque integral en salud oral que garantice acceso equitativo a tratamientos, dignidad y calidad de vida, reconociendo su vínculo directo con la salud mental.

## Abstract (en)

The article analyzes how tooth loss among older adults is not merely a clinical issue but a social and emotional phenomenon marked by shame, stigma, and structural inequality in oral health in Chile. From a qualitative, narrative approach with a descriptive scope and a hermeneutic (interpretive) orientation, 12 interviews were conducted in the commune of Cerro Navia, in the Metropolitan Region of Chile, between August and September 2024. The narratives reveal that tooth loss affects self-esteem, identity, and social participation. Shame manifests in behaviors such as covering the mouth, avoiding smiling, speaking in public, or attending social gatherings, leading to social isolation and psychological suffering. The testimonies show that lack of financial resources and limited access to public dental care exacerbate this distress, forcing many individuals to resign themselves to tooth loss. This condition, in addition to limiting nutrition and everyday functioning, becomes a social marker of class, reinforcing moral judgments associated with poverty and poor hygiene. The study concludes that shame cannot be understood solely as an individual emotion, but rather as a social device that reproduces inequalities and exclusions. An integrated approach to oral health is proposed, ensuring equitable access to treatment, dignity, and quality of life, while recognizing its direct link to mental health.

## Introducción

La vergüenza ha sido reconocida históricamente como una de las emociones humanas fundamentales, que constituye un elemento clave en la configuración de las relaciones sociales^1^. Esta noción se ha conceptualizado como una emoción social por excelencia, abordada desde diversas disciplinas, particularmente la sociología, la cual ha explorado su dimensión intersubjetiva y estructural a partir de enfoques teóricos provenientes de autores como Simmel^2^, Elias^3^ y Goffman^4^. 

En el ámbito de la salud, la vergüenza ha cobrado especial relevancia en las investigaciones vinculadas a la salud mental, siendo objeto de análisis en múltiples estudios que examinan su impacto en la vivencia del sufrimiento psíquico y en el acceso a servicios de atención^5^^,^^6^^,^^7^^,^^8^^,^^9^^,^^10^^,^^11^. De igual modo, la literatura psiquiátrica ha profundizado en las relaciones entre salud mental, cultura y vergüenza, destacando cómo esta emoción es modulada por normativas culturales que condicionan tanto su expresión como su interpretación clínica^12^^,^^13^^,^^14^.

Jean-Paul Sartre^15^ sostenía que la vergüenza surge cuando un individuo se percibe a sí mismo como deshonroso a través de la mirada del otro. Uno de sus ejemplos clásicos ilustra esta idea mediante la figura de un hombre que realiza un gesto vulgar y, al percatarse de que ha sido observado, experimenta vergüenza. Esta emoción, profundamente arraigada, es omnipresente y suele manifestarse desde edades muy tempranas. Sin embargo, a pesar de su aparente familiaridad, persisten debates en torno a su definición y funcionamiento^1^^,^^16^.

La vergüenza asociada al estigma tiende a mantenerse oculta en la experiencia social. Diversos autores han sostenido que esta emoción constituye un verdadero tabú en la cultura contemporánea. Goffman^6^ nos dirá que el estigma opera como un atributo desacreditador que transforma a una persona de un individuo completo y normal en uno disminuido y desacreditado, generando una fractura entre la identidad real y la identidad social asignada. Esta discrepancia produce vergüenza porque expone al sujeto a la mirada evaluadora del otro, quien confirma la marca que deteriora su valor social.

Los planteamientos de Scheff^17^, Kaufman^18^ y Brown^19^ convergen en señalar que la vergüenza, pese a ser una emoción universal y profundamente enraizada en la vida social, permanece sistemáticamente invisibilizada. Scheff^17^ destaca que la vergüenza, aunque oculta, es la emoción más frecuente en las sociedades modernas; Kaufman^18^ enfatiza que existe un tabú cultural que nos lleva a actuar como si la vergüenza no existiera; y Brown^19^ añade que, incluso en ámbitos profesionales y académicos se evita tratarla abiertamente. En conjunto, estos autores muestran que la vergüenza, aunque omnipresente, queda marginada del discurso social, dificultando su reconocimiento, análisis y abordaje.

En ese sentido, y respecto a la salud oral, David Le Breton^(20,21,22, 23,24)^ nos dirá que la boca no es solo un conjunto funcional de piezas dentarias, sino un espacio cargado de valor simbólico, en otras palabras, es lugar de expresión, de deseo, de placer y de dignidad. La pérdida dental, por tanto, afecta no solo la masticación, sino también la autoestima, la imagen corporal y la interacción social, generando sentimientos de vergüenza o incomodidad, especialmente en espacios públicos o laborales. De esta forma, la comprensión de la vergüenza en salud oral puede profundizarse si asumimos, como plantea Le Breton^25^, que el rostro es el núcleo simbólico donde la identidad se hace visible y vulnerable. Su carácter enigmático y siempre expuesto convierte cualquier modificación, incluida la pérdida dental, en una alteración que desestabiliza la relación entre el sujeto y la mirada ajena. En esta línea, la vergüenza emerge cuando el rostro deja de corresponder al “rostro de referencia” que sostiene la autoestima y la continuidad de sí. De esta manera, más que un fenómeno clínico, la pérdida dental se vuelve una herida en la rostridad, ese espacio donde se inscriben la dignidad, la diferencia y el reconocimiento social. Esta perspectiva permite situar el estigma dental como una afectación directa del rostro, es decir, del lugar donde la cultura asigna valor, moralidad y humanidad.

Esto nos permite comprender el objeto de este estudio, ya que la pérdida dental no solo limita la alimentación o el habla, sino que hiere la identidad y la dignidad del sujeto. Es aquí donde el estigma, desde la perspectiva gofmanniana, cobra una potencia especial, ya que no se trata solo de una carencia física, sino de una transformación visible que reconfigura el rostro, la expresión emocional y la relación con los otros^6^. Ello nos permite entender la vergüenza como dimensión central del estigma dental, donde el cuerpo queda expuesto al juicio social.

### Vergüenza, Estigma y Salud Oral

Diversas investigaciones han evidenciado que la salud oral influye de manera significativa en la calidad de vida durante la vejez, incidiendo no solo en la funcionalidad alimentaria, sino también en dimensiones psicosociales como la autoestima y la participación en espacios comunitarios^26^^,^^27^. En este marco, la vergüenza adquiere un papel preponderante como emoción autorreferida que emerge ante la percepción de juicio negativo, generando sentimientos de insuficiencia personal. Esta emoción ha sido vinculada a consecuencias como el retraimiento social, la ansiedad y síntomas depresivos, lo cual agrava la carga emocional de las personas mayores que experimentan pérdida dental u otros problemas orales visibles^28^^,^^29^. En esa línea, Tsironis^30^ identifica la vergüenza como variable mediadora entre la salud oral y el malestar psicológico en adultos mayores. Sin embargo, también afectaría a las poblaciones infantiles ya que, desde edades tempranas, niños y niñas están atentos a su apariencia y a la percepción que otros tienen de ellos, y pueden experimentar vergüenza si perciben que no cumplen con ciertos estándares estéticos socialmente valorados^31^^,^^32^.

No obstante, en la vejez, la salud oral y, en particular, la pérdida dental tiene una significación importante, que impacta en la socialización, alimentación, entre otras áreas. Un estudio de Ferreira^33^ muestra que la pérdida dental es vivida como un proceso naturalizado, asociado a la edad, al descuido personal y a condiciones estructurales como la pobreza y el difícil acceso a tratamientos odontológicos. 

El fenómeno de la pérdida dental como un problema prevalente, especialmente en países en desarrollo, afecta de forma desproporcionada a personas de bajos ingresos y niveles educativos precarios^34^^,^^35^. De esa manera, la salud bucal refleja desigualdades sociales más amplias, vinculadas a trayectorias de vida marcadas por la pobreza, la precariedad laboral, el limitado acceso a servicios de prevención y la desinformación^36^^,^^37^.

De ese modo, podemos entender la vergüenza como una emoción social compleja y autorreferida, que puede desencadenar comportamientos de evitación, retraimiento o agresividad y estigmatización^38^^,^^39^. En esa línea, uno de los principales problemas que se presentan con la salud oral es la estigmatización, específicamente, por la pérdida de dentadura. Ya Goffman^6^ nos decía que el estigma producía vergüenza y esta no es solo una emoción interna, sino un efecto relacional que emerge del temor a ser descubierto, juzgado o reducido a la característica estigmatizada. En contextos donde la apariencia corporal adquiere peso moral -como ocurre con la salud oral- el estigma se intensifica y la persona aprende a gestionar su visibilidad mediante estrategias de ocultamiento, evitación o silencio, buscando protegerse de esa desvalorización pública. De acuerdo a Doughty^40^ se trata de un fenómeno común, pero poco definido y abordado en la literatura científica. Como consecuencia, el estigma relacionado con la salud oral se entiende como un proceso social que desvaloriza a las personas por condiciones visibles de su salud bucal (como pérdida dental, caries avanzadas o malformaciones), atribuyéndoles rasgos negativos como dejadez, pobreza o falta de higiene. Este estigma se expresa a través de juicios personales, discriminación estructural, autoestigmatización y barreras para el acceso a la atención odontológica^41^.

Según el análisis de Malik^42^, las personas que experimentan estigma relacionado con su salud bucal y su pérdida dental tienden a internalizar estos juicios, culpándose a sí mismas por no haber prevenido el deterioro, aunque las causas estén vinculadas a determinantes estructurales como pobreza, falta de educación en salud o acceso limitado a servicios. Esta culpa internalizada se transforma en evitación activa, como se ha observado en estudios similares^40^^,^^43^. Por otro lado, Andersen, Varga y Folker^44^ señalan que el estigma opera como una construcción que se ancla en normas sociales dominantes y se manifiesta en prácticas institucionales, lo que coincide con las experiencias de las personas participantes que evitaron consultas odontológicas por miedo al juicio clínico. Este tipo de estigma internalizado, tal como lo conceptualiza Pinel y Bosson^45^, afecta la autopercepción y desencadena mecanismos de autoevitación, agravando los efectos del descuido bucal. Khalid y Quiñonez^46^ introducen la noción de “dientes blancos y rectos” como un prerrequisito simbólico de ciudadanía estética, lo que refuerza la idea de que las condiciones dentales no son neutrales, tal como ya indicaba anteriormente Le Breton^25^, sino evaluadas desde cánones normativos que legitiman o excluyen. Este simbolismo se traduce en experiencias de exclusión social narradas por los participantes del estudio, que reportan haber sido objeto de burla o juicios morales por su apariencia dental.

## Metodología

Desde un enfoque cualitativo de tipo narrativo, con un alcance descriptivo y una orientación hermenéutica (interpretativa), se realizaron 12 entrevistas en profundidad con preguntas no estructuradas, siguiendo un enfoque temático basado en las propuestas de Köehler Riessman^47^ y Chase^48^, que permite obtener un entendimiento profundo de las experiencias de los participantes. 

Las entrevistas fueron realizadas por un profesional de las ciencias sociales, posterior a la atención clínica de seguimiento que realizaba la cirujana dental. Estas se llevaron a cabo en el dispositivo de Atención Primaria Dr. Steeger, ubicado en la comuna de Cerro Navia, una zona caracterizada por altos niveles de vulnerabilidad socioeconómica en la Región Metropolitana de Chile. El trabajo de campo se realizó entre agosto y septiembre de 2024.

Los participantes fueron adultos mayores, con edades entre 70 y 80 años, que habían recibido el “Examen dental preventivo del adulto mayor” (EDePAM) entre 2020 y 2022. El EDePAM es una herramienta de evaluación odontológica de Chile enfocada en personas mayores de 60 años. Su objetivo es clasificar el riesgo de enfermedades bucales para mejorar la calidad de vida, complementando el examen médico preventivo (EMPAM) y facilitando el acceso al primer nivel de atención. La selección se realizó mediante un muestreo intencionado, eligiendo a quienes participaron en dicha intervención. En este estudio, se alcanzó la saturación al analizar las intersecciones entre la vergüenza y el bienestar psicológico de las personas tratadas con el EDEPAM. Tras las 12 entrevistas en profundidad en las que se abordaron las experiencias de los participantes, las categorías analizadas mostraron una consistencia significativa, lo que permitió concluir que el fenómeno había sido explorado de manera integral.

La investigación contó con la aprobación del Comité de Ética de la Facultad de Odontología de la Universidad de Chile (código 02/22) y el Comité ético científico del Servicio de Salud Metropolitano Norte, en la Región Metropolitana en Chile (código 28/2022). Se utilizó un consentimiento informado para resguardar el anonimato de los participantes. Las entrevistas fueron transcritas y posteriormente analizadas mediante el software ATLAS.ti, utilizando un enfoque narrativo con una aplicación temática^47^^,^^48^.

## Análisis: La vergüenza por no tener dientes

La vergüenza, como emoción, descrita por Ahmed^49^, no solo afecta profundamente al yo, sino que se encarna en el cuerpo y se ve moldeada por las interacciones sociales^50^. En los relatos de esta investigación, esta emoción surge como una respuesta a la ausencia de una dentadura o una adecuada salud oral, evidenciando una conexión entre el cuerpo, la identidad y las dinámicas de exclusión social.

Para los entrevistados de esta investigación, la vergüenza se manifiesta físicamente con el acto de cubrirse la boca, evitar reírse o incluso hablar en público: “*A mí me daba vergüenza. Entonces no podía conversar porque es como que se me enredaba la lengua*” (E1). Esta acción refleja cómo el cuerpo, al ser objeto de juicio social, se convierte en el punto central de esta emoción. Esto se puede ver en trabajos que requieren interacción con otros, en los que la vergüenza se intensifica, como lo relata una entrevistada:

“*Me daba vergüenza, y tuve un trabajo donde mi jefa me decía, en dos oportunidades, que no tener tu dentadura era muy antihigiénico, y yo era la cocinera*”. (E6)

Este juicio externo refuerza la percepción de insuficiencia personal y profesional, pero también un sufrimiento social respecto a la vergüenza y el estigma^6^^,^^25^.

La identidad también se ve profundamente afectada y deteriorada^6^. La vergüenza, al estar ligada a cómo se perciben a sí mismos, lleva a las entrevistadas a sentirse inferiores: “*Yo me sentía como que no era nadie, era una cosa así, como que diferente. No es igual que con las personas que tienen sus dentaduras bonitas*” (E1), señala una persona, destacando cómo esta experiencia daña su autoestima, estigmatizándola y produciendo vergüenza. Como decía Le Breton^25^, el rostro deja de corresponder al “rostro de referencia” que sostiene la autoestima y la continuidad de sí, pasando a ser un rostro de sufrimiento que se expresa en lo psíquico y lo social. De esta forma, la ausencia de dentadura no solo representa una limitación funcional, sino que también se convierte en un potente símbolo de carencia y vulnerabilidad, que afecta profundamente la percepción que las personas tienen de sí mismas. Este hecho actúa como un catalizador que refuerza ideas negativas sobre su propio valor y autoestima, intensificando una sensación de insuficiencia personal. En este contexto, los estereotipos sociales juegan un rol fundamental al amplificar esta experiencia emocional, como lo ilustra una entrevistada:

“*Es una vergüenza, uno se siente inferior. Me están criticando por esto y es verdad. No tengo el tiempo o la cantidad de dinero para ir a solucionar este problema de mis dientes* […] *A uno lo entristece que se rían de uno. Es lo peor que te puede pasar. Y uno tiene que morderse ese sentimiento que uno va generando*”. (E4)

Aquí se evidencia cómo el juicio externo no solo hiere, sino que también se internaliza, arraigándose en las personas como una autoevaluación negativa que las deja atrapadas en un ciclo de vergüenza y desesperanza y, por ende, con implicancias sociales. De manera similar, otro entrevistado describe cómo la percepción estigmatizante de otros genera un sentimiento de indignidad y desvaloración: “*Una persona que le faltan los dientes, es como que estuviera cochina o no sé, una cosa sucia. Claro, esto es lo que siente uno*” (E1). Estas palabras reflejan no solo el peso de los estigmas sociales, sino también la forma en que estas ideas son asumidas como verdades por quienes las experimentan, profundizando las heridas emocionales y aumentando el impacto psicoemocional de la situación, que afectan su salud mental.

En ambos casos, se evidencia que el daño no radica únicamente en los comentarios o actitudes de los demás, sino en cómo estas percepciones se filtran hacia la identidad de quienes las padecen, marcándolos con un sentimiento de vergüenza constante y una sensación de inadecuación que resulta difícil de superar.

La vergüenza también genera una exclusión social significativa. Las personas tienden a evitar situaciones donde puedan ser vistas o juzgadas por los demás: “*Me invitaban a cenar y me daba vergüenza, porque todos compartiendo me dirían, ya sírvete mujer. ¿Qué iba a hacer si no tenía dientes? Mejor no iba*” (E10). Este aislamiento va más allá de evitar eventos sociales, limita las oportunidades de conexión humana y aumenta el sentimiento de soledad, malogrando el bienestar psicoemocional de las personas. En esa línea otra entrevistada explica: “*Resulta que yo tenía que ir con mi hijo del brazo para la iglesia cuando se casó. Y yo no tenía dientes. ¿Se imagina la pena que tenía?*” (E8). Este comentario ilustra cómo la vergüenza afecta la vida cotidiana, exacerbando sentimientos de tristeza y exclusión social, tal como Le Breton^25^ planteaba acerca la “herida en la rostricidad”.

Las condiciones estructurales juegan un papel crucial en perpetuar esta experiencia. La falta de recursos económicos limita la capacidad de las personas para solucionar el problema, intensificando el ciclo de vergüenza*.* Para algunos, la precariedad económica significa resignarse:

“*Porque los dientes picados o que te falte uno, hace que andes tapándote la boca, no puedes conversar, porque te da vergüenza. Y si no tienes plata para arreglarte, tienes que seguir así no más*”. (E2)

Esta resignación refuerza la sensación de estancamiento y aislamiento social provocada por la carencia y desigualdad social en el acceso público a una salud oral digna. 

Por último, la vergüenza no solo afecta la relación con las demás personas, sino también consigo misma, limitando la posibilidad de proyectarse de manera positiva. Otro entrevistado lo resumía así: “*Yo estaba bastante avergonzado porque se me iban cayendo los dientes y uno en esos años andaba pololeando sin dientes. Un diente por acá, otro por allá*” (E3). En este sentido, la vergüenza no es solo un sentimiento, sino un reflejo de desigualdades sociales y estructurales que afectan profundamente la vida de las personas^6^^,^^25^.

### Vergüenza y aislamiento social

El aislamiento social es una consecuencia significativa y compleja de la vergüenza, que no solo refleja un sufrimiento psíquico profundo, sino que también perpetúa una desconexión que afecta la calidad de vida y las relaciones interpersonales, en definitiva, un sufrimiento social. Según Caponi y Kozuchovski^51^, la vergüenza se relaciona intrínsecamente con trastornos como la depresión y la ansiedad, afectando la manera en que las personas enfrentan la interacción social y perciben su lugar en la comunidad. Este vínculo emocional, observado en los testimonios analizados, revela que la vergüenza no se limita a una experiencia interna, sino que también modela comportamientos que refuerzan el aislamiento y la exclusión autoimpuesta.

La vergüenza actúa como un motor de evasión social, limitando las oportunidades de interacción y conexión. Una entrevistada describe esta dinámica de manera explícita:

*“Pero cuando tenía que salir, estar con más gente, ahí me daba vergüenza, ahí yo me tapaba la boca* […] *No salía a la calle porque me daba vergüenza*”. (E1)

Este acto de evitar espacios públicos es una respuesta al temor de ser juzgada, ilustra cómo la vergüenza se inscribe en el cuerpo y moldea las decisiones cotidianas, es la violencia simbólica de la que Le Breton hace referencia, ya que ese sufrimiento no brota del cuerpo por sí mismo, sino más bien de la trama social, que interpreta, mira, clasifica y rechaza^25^. 

En contextos más íntimos, esta emoción también inhibe la capacidad de participar y disfrutar de experiencias compartidas, como señala otra entrevistada:

“*Es que no podía comer. Tenía que irme a comer escondidas* […] *Veía a todos que estaban comiendo y no podía comer porque no tenía dientes ni muelas*”. (E2)

Este comportamiento no solo refleja un temor al juicio externo, sino también una sensación de inferioridad internalizada que se refleja en ese “imán de miradas y comentarios” que señala Le Bretón^25^ y que, por tanto, aísla a la persona.

El temor al rechazo o la burla refuerza esta autoexclusión, que intensifica un ciclo de aislamiento. Como lo expresa otro testimonio: “*Esto es como que uno se aísla. Se aísla porque ¿qué saco con ir? Hay personas que después se ríen de uno*” (E6). Las invitaciones a eventos sociales, en lugar de ser espacios para la conexión, se convierten en fuentes de ansiedad y estrés: “*Me invitaban a tomar once* [la merienda]*, me daba vergüenza* […] *No iba*” (E10). Este patrón de evitación social se acompaña de estrategias de protección que refuerzan la desconexión, como el uso de mascarillas para minimizar el contacto: “*No, salía pero me ponía mascarilla. Cuando salía, salía sola, por ejemplo, yo iba a una plaza, me sentaba sola. No me juntaba con nadie*” (E10). En este contexto, el aislamiento social opera como un mecanismo defensivo que, aunque busca proteger al individuo de la vergüenza y el rechazo de no tener dientes, tiene consecuencias negativas a largo plazo. Refuerza el sufrimiento emocional, debilita los vínculos sociales esenciales y profundiza la sensación de soledad. Así, el aislamiento social, al perpetuarse, se convierte en un ciclo difícil de romper, evidenciando la necesidad de abordar la vergüenza no solo como un problema emocional, sino también como una barrera estructural y social.

### Sufrimiento psíquico por no tener dientes

El sufrimiento psíquico es una experiencia subjetiva que se caracteriza por la percepción de sentimientos negativos intensos y cambios en el yo. Puede ser causado por la angustia, la culpabilidad, la pérdida, el dolor, el temor, la preocupación, la frustración e incluso la sumisión^52^. De acuerdo con Amorim^53^, estaría relacionado con la presión a la que un sujeto está sometido por parte de las relaciones sociales, como las instituciones con las que se vincula, que crean malestar y sufrimiento. En los testimonios analizados, el sufrimiento psíquico emerge como una consecuencia directa de la vergüenza asociada a la condición dental. La necesidad de ocultar este aspecto físico genera tensiones emocionales constantes:

“*Y lo negativo era que tenía que esconderme y dar una sonrisa falsa. ¿Por qué? Para no amargarme. Porque si yo me amargaba era peor y más se iban a burlar de mí*”. (E4)

Aquí se observa cómo la vergüenza impulsa un esfuerzo por disimular el malestar y el estigma social, reforzando un estado interno de angustia y desgaste emocional. La dificultad para participar plenamente también intensifica este sufrimiento, instalándolo en un lugar social. 

Otra de las personas describe cómo la condición dental afecta su capacidad de expresarse, alimentando sentimientos de rechazo y opresión:

“*Cuando uno tiene cierta dificultad y los dientes se le van cayendo, uno tiene que rechazarse y aguantarse de varias cosas. Aunque quiere opinar en algún tema, no puedo expresarla, porque no se van a reír de lo que uno dice, sino de lo que ellos ven en la boca* […] *He sufrido, como le digo, mucho por la dentadura*”. (E6)

Este testimonio evidencia la forma en que el sufrimiento psíquico resulta de una autocensura impuesta por el miedo al juicio externo. La experiencia del sufrimiento también se manifiesta físicamente, como un recordatorio constante de la vergüenza y el aislamiento. Un entrevistado expresa cómo el mal aliento y las carencias dentales intensifican su sentimiento de inferioridad:

“*Yo siempre trabajé de mozo en los casinos, de bancos, de empresas, y atendía siempre la jefatura mayor, los dueños, visitas que invitaban, y yo tenía que atenderlos. Entonces ahí trataba de esquivar, no estar muy cerca de las personas.* […] *me tenía que preocupar harto del aseo antes de la hora en que había que servir el almuerzo. Tenía que hacerme un aseo rápido a veces, y me echaba una pastilla de menta, por último, en la boca para no tener ese mal aliento. Me sentía disminuido, ¿sabe? Me sentía totalmente avergonzado*”. (E7)

La necesidad de implementar estrategias temporales, como el uso de pastillas para el aliento, no resuelve el problema subyacente, sino que refuerza la percepción de insuficiencia personal. En casos más severos, este sufrimiento se transforma en depresión, combinando tristeza, enojo y desesperanza:

“*Yo estaba triste, y lloraba, porque se me caían mis dientes solos y sanos, sin picadura, y era joven* […] *No sé, pues me daba pena. No tenía medios yo como para pagar particular y rápido, porque es muy carísimo*”. (E8)

Este testimonio ilustra cómo las limitaciones económicas intensifican el malestar psicológico, creando una espiral de impotencia y frustración.

Finalmente, el sufrimiento psíquico se amplifica en las interacciones sociales cotidianas, como al compartir comidas con amigos: “*Me sentía súper mal, horrible* […] *Usted sabe que no puede comer lo que sus amigos están comiendo. Por ejemplo, maní, una manzana o carne asada*” (E10). Estas limitaciones refuerzan un sentido de exclusión y una pérdida de la conexión social esencial para el bienestar emocional. En conjunto, los testimonios muestran cómo la vergüenza y el aislamiento social convergen para generar un sufrimiento psíquico que afecta múltiples dimensiones de la vida, desde la autoimagen hasta la interacción con el entorno, perpetuando un estado de malestar que exige ser abordado desde perspectivas tanto individuales como estructurales.

### Perdiendo la calidad de vida

Hablar de calidad de vida puede referirse a un estado de salud, funcionamiento físico como también a un estado de bienestar y felicidad^54^. La pérdida dental afecta significativamente la calidad de vida, entendida como un estado que integra salud, bienestar físico, emocional y social. Esta condición, al entrelazarse con la vergüenza, el aislamiento social y el sufrimiento psíquico, deteriora la capacidad de las personas para participar plenamente en la vida cotidiana. Según Aytac^55^^,^^56^, el bienestar psicológico y emocional es clave para enfrentar el estrés, la tensión y mantener la autonomía personal. Sin embargo, los testimonios analizados muestran cómo estas dimensiones quedan comprometidas, generando una experiencia de vida marcada por la frustración, la inseguridad y la exclusión.

La pérdida dental limita directamente la capacidad de masticar, lo que restringe la dieta y genera incomodidades persistentes. Un entrevistado expresa claramente esta limitación: *“Uno no puede comer carne asada o cuestiones más o menos duras como los cereales, cuestiones de esas. Es complicado, es muy difícil*” (E3). Estas restricciones alimentarias no solo privan a las personas de disfrutar ciertos alimentos, sino que también refuerzan sentimientos de impotencia: “*Para comer es complicado. Una, tienen que andar con un cuchillo comiendo pedacitos y tragándoselos, moliendo la comida a escondidas*” (E5). 

Las adaptaciones constantes transforman lo que debería ser una experiencia placentera en un recordatorio diario de la limitación física. Además, situaciones humillantes como la pérdida de prótesis dentales en público intensifican estas dificultades: “*Fuimos a la costa, andábamos con un grupo de adultos mayores* […] *y estornudé tan fuerte que la prótesis salió volando, me llené de vergüenza*” (E5). Estos eventos no solo dificultan el acto de comer, sino que también generan temor a situaciones similares, limitando aún más la interacción social. 

Las limitaciones físicas asociadas con la pérdida dental están acompañadas de profundas implicaciones emocionales y sociales. La vergüenza y el estigma se convierten en una barrera significativa para la interacción social, afectando el bienestar emocional de manera directa: “*Entonces, hay vergüenza* […] *uno al sonreírse se ve mal. Se ve muy mal*” (E6). Este sentimiento de vergüenza lleva a las personas a reprimir su expresión personal, como lo expresa la misma entrevistada: “*Uno se siente mal. Si estás, por ejemplo, en un grupo, te da no sé qué conversar, mejor me callo*” (E6). 

La autocensura no solo limita la comunicación, sino que también refuerza un ciclo de inseguridad, en el que las personas prefieren evitar exponerse al juicio de los demás. Este sentimiento de vergüenza está estrechamente ligado a problemas como el mal aliento, que intensifica la sensación de incomodidad personal: “*La autoestima andaba bajísimo. Porque, como le digo, a veces… el mal aliento se sentía. Yo vivía acomplejado*” (E7). Las experiencias refuerzan una percepción negativa del propio cuerpo, afectando profundamente la autoconfianza. Actividades simples, como comer una manzana, se ven condicionadas por esta situación: “*Por ejemplo, para comerme una manzana tengo que tener mucho cuidado* […] *no quiero perder mis dientes, saco lo que tengo para tragar no más*” (E7).

Estas restricciones perpetúan un sentimiento de exclusión que afecta la percepción del propio valor y la capacidad de disfrutar la vida cotidiana. Este sufrimiento se expresa en sentimientos de tristeza y desesperanza, en detraimiento de su bienestar psicológico y social como lo describe una entrevistada:

“*Yo estaba triste, porque no quería salir con mi esposo, él está muerto ahora, y no aproveché mi vida saliendo juntos, yo sé que en ese momento estaba deprimida porque no tenía dientes*”. (E8)

La incapacidad de acceder a tratamientos dentales debido a los altos costos exacerba este malestar, generando un ciclo de frustración e impotencia. La falta de recursos económicos no solo limita las opciones de tratamiento, sino que también intensifica el sentimiento de desesperanza:

“*Claro, imagínese iba quedando de a poco sin dientes* […] *no había nada de plata para arreglármelos, entonces, claro, y los dentistas en ese tiempo eran muy costosos*”. (E3)

Así se evidencia cómo las barreras económicas agravan el sufrimiento psíquico, refuerzan el ciclo de exclusión y deteriora la calidad de vida.

La rabia y la humillación son también emociones frecuentes en esta experiencia, como lo describe otro entrevistado: “*Como se reían de mí por no tener dientes y no lograr comer, sentía rabia, mucha rabia*” (E10). Aunque esta rabia puede inicialmente dirigirse hacia otros, también tiene un impacto interno, afectando la autoestima y reforzando sentimientos de insuficiencia personal. Este tipo de sufrimiento no solo limita el bienestar psicológico, sino que también socava la capacidad de las personas para proyectarse de manera positiva en sus interacciones sociales y personales, en definitiva, pierde su calidad de vida.

La intersección entre la pérdida dental, la vergüenza y el sufrimiento psíquico configuran un panorama de deterioro significativo en la calidad de vida. La incapacidad de disfrutar de alimentos, participar plenamente en interacciones sociales o expresarse libremente afecta no solo el bienestar físico, sino también la percepción de autonomía y felicidad.

En esa línea, una de las entrevistadas expresa: “*Sí, me aguantaba* […] *porque solo podía tragar*” (E8), evidenciando cómo estas limitaciones se convierten en una carga diaria que compromete tanto el cuerpo como las emociones. Estos factores no solo afectan la salud física, sino que también tienen un impacto profundo en la percepción de uno mismo y en la relación con los demás. Le Bretón señala que el sufrimiento emerge no solo del daño físico, sino de la trama social que interpreta ese cuerpo como problemático y lo somete a miradas persistentes, incomodidad y exclusión, convirtiendo al rostro alterado en un signo que activa una forma de sufrimiento social.

En conjunto, los testimonios muestran que la pérdida dental no es solo un problema médico, sino también una cuestión que afecta profundamente el bienestar emocional, psicológico y social. Abordar esta problemática requiere no solo soluciones clínicas, como el acceso a tratamientos dentales asequibles, sino también un enfoque integral que considere las implicaciones psicológicas y sociales. Es crucial buscar estrategias que no solo restauren la funcionalidad, sino también la dignidad y la calidad de vida de quienes enfrentan esta situación. De este modo, sería posible reducir el impacto de la vergüenza, el aislamiento y el sufrimiento, promoviendo un bienestar más pleno e inclusivo para las personas afectadas.

### Acceso a la salud oral y desigualdad estructural

La dificultad de acceso a la salud dental en Chile se manifiesta como un problema estructural en el que convergen factores económicos, sociales y territoriales que profundizan la desigualdad. Si bien existe el Plan Nacional de Salud Bucal 2021-2030, este prioriza a los menores de edad y no a la población en situación de vejez. El EDEPAM es una herramienta aun no instalada en Chile, por lo que la población objetivo de este estudio no tuvo acceso. En este marco, los testimonios recogidos revelan que la atención odontológica no se percibe como un derecho garantizado por el Estado chileno, sino como un privilegio condicionado al poder adquisitivo. En palabras de una entrevistada:

“*Porque casi en ninguna parte te ve el dentista sin pagar. Entonces en ese tiempo que uno era pobre no tenía los medios como para presentarme a un dentista particular, y así perdí los dientes*”. (E1)

Esta afirmación ilustra con claridad cómo el acceso a los servicios dentales queda restringido a quienes poseen recursos, mientras los sectores más empobrecidos deben optar por la resignación o la pérdida progresiva de piezas dentales. El costo elevado de los tratamientos ha constituido la principal barrera de acceso a la salud oral. La imposibilidad de costear procedimientos como prótesis o extracciones genera una experiencia de exclusión reiterada en los relatos. Como señala otra entrevistada:

“*Es una barrera grande acceder al dentista, porque lo intenté* […] *y la pura pieza me salía como 100 mil pesos. En Chile la dentadura y todas estas cosas son muy caras*”. (E3)

La dimensión económica convierte la salud bucal en un privilegio, inaccesible para la mayoría, y coloca a las familias en la disyuntiva de priorizar entre la alimentación, la educación de los hijos o la atención dental. En este sentido, los testimonios no solo muestran la imposibilidad de costear servicios, sino también la interiorización de una jerarquía de necesidades, donde la dentadura queda relegada al último lugar. Así lo expresa otro entrevistado:

“*No cuidamos nunca la dentadura. Yo de joven tuve mi dentadura mala. Había y hay otras prioridades, y siempre los dentistas han sido caros, así perdí casi todos mis dientes*”. (E9)

El problema, sin embargo, no se limita al ámbito económico. Existen también diferencias marcadas en el acceso territorial, que refuerzan la sensación de abandono de quienes viven en sectores rurales. Una entrevistada afirma: “*Sobre todo para la gente que vive en el campo, los dentistas no llegan allá, están muy abandonadas esas personas*” (E1). De ese modo, la precariedad no solo se mide por la capacidad de pago, sino también por la ubicación geográfica. La salud oral en zonas rurales aparece como una dimensión prácticamente inexistente, donde la presencia del Estado es mínima y, cuando existe, se materializa en programas puntuales, muchas veces insuficientes y asistencialistas. Así lo narra otra entrevistada:

“*Llegaba siempre los días miércoles una ambulancia* […] *era un programa a través del Estado, me parece, y ahora que lo veo en retrospectiva, eso no era suficiente*”. (E7)

Estos operativos, aunque bien valorados, refuerzan la idea de que la atención oral pública se ofrece de manera esporádica y paliativa, y no como un derecho permanente.

Otro aspecto que emerge con fuerza en los relatos es la insuficiencia del sistema público. Los consultorios se describen como espacios que ofrecen lo mínimo y que obligan a las personas a esperar largos periodos de tiempo para recibir atención básica. Uno de los entrevistados comenta:

“*En el consultorio no existía esto de arreglar la dentadura* […] *en Chile la dentadura es muy cara, arreglarse los dientes siempre era algo que uno debía hacer de forma particular. Eso debería ser público*”. (E6)

La ausencia de tratamientos reconstructivos y la falta de cobertura en prótesis refuerzan la desigualdad, puesto que la salud bucal es tratada como un ámbito secundario frente a otras necesidades médicas. En esa misma línea el relato de otro entrevistado evidencia la frustración con la burocracia y los plazos del sistema:

“*Más de un año se demoraron en atenderme porque el tratamiento en lo público es muy lento, hay que saber esperar, pedir las horas y todo eso y, aun así, a veces hay servicios que no se prestan y uno debe ir a lo particular, siempre y cuando tengas dinero*”. (E8)

Este tipo de experiencias genera un círculo de desprotección en el cual la espera se convierte en pérdida, en donde las piezas dentales se deterioran más rápido de lo que el sistema es capaz de responder. La percepción de calidad diferenciada entre lo público y lo privado también ocupa un lugar central en las narrativas. Mientras que las atenciones particulares se consideran mejores y de mayor dedicación, el sistema público es percibido como lo último, como una opción de baja calidad a la que se recurre únicamente por necesidad. En palabras de una entrevistada:

“*Las atenciones pagadas en el dentista son más bonitas, son mejores, atienden mejor* […] *en el consultorio es como lo último, a uno no lo tratan tan bien*”. (E6)

Esta diferencia simbólica refuerza la mercantilización de la salud, donde la dignidad y el cuidado dependen directamente del pago, instalando una jerarquización que naturaliza la exclusión y que transforma la atención dental en un marcador social de clase. 

Frente a estas dificultades, las personas elaboran estrategias de afrontamiento que oscilan entre el sacrificio económico, la búsqueda de apoyo familiar y la resignación. Algunos entrevistados relatan cómo los hijos intentaban reunir dinero para pagar consultas privadas:*“Mi hijo mayor me decía, a ver si juntamos para ir a tal parte, averiguó en varias partes, pero la situación económica no me acompañaba*” (E8). Estas frases expresan cómo el acceso a la salud dental se convierte en una carga emocional y económica que rebasa al individuo y se comparte en el núcleo familiar, muchas veces sin éxito.

En definitiva, los relatos analizados permiten afirmar que el acceso a la salud dental en Chile está atravesado por la mercantilización, la desigualdad territorial y la insuficiencia del sistema público. La salud bucal se configura como un espacio de desigualdad estructural, donde los costos inaccesibles, las listas de espera y la percepción de un Estado ausente refuerzan la exclusión. La dentadura, más que un aspecto estético, se convierte en un marcador social que distingue a quienes pueden costear la mantención de una sonrisa sana de quienes deben resignarse a la pérdida y tal como se ha visto hasta acá, la vergüenza. Esta situación no solo afecta la funcionalidad y la salud física, sino también la autoestima, el bienestar emocional y la percepción de dignidad. De este modo, la salud dental se revela no como un ámbito secundario, sino como un espacio crítico desde el cual se reproducen las desigualdades sociales y se cuestiona de manera directa el carácter de derecho universal que debería tener la salud.

## Discusión

La vergüenza asociada a la pérdida dental se configura como una experiencia compleja que impacta tanto la salud mental como el bienestar psicoemocional y social de las personas afectadas. En este análisis, la vergüenza se muestra como una emoción profundamente enraizada en el cuerpo, moldeada por las interacciones sociales y amplificada por las desigualdades estructurales. Tal como lo describe Ahmed (49), la vergüenza no solo afecta la percepción del yo, sino que también influye en cómo las personas interactúan con su entorno, marcándolas con una sensación de insuficiencia y exclusión que se refuerza con el tiempo. En esa misma línea, y tal como planeta Le Bretón^25^, el sufrimiento no puede comprenderse únicamente como una vivencia íntima, sino como una experiencia radicalmente social, producida por la mirada, el juicio y la posición que el cuerpo ocupa en el mundo.

Los testimonios analizados revelan cómo esta emoción no solo se internaliza, sino que se manifiesta en conductas que limitan la expresión y la interacción social. La necesidad de ocultar la falta de dientes, de evitar reírse o participar en conversaciones, refleja el juicio social que se inscribe en el cuerpo, generando actitudes defensivas frente al estigma. Este fenómeno, observado en relatos como el de una entrevistada que evitaba reírse y prefería el silencio, evidencia a la vergüenza como un sentimiento que restringe la participación plena en la vida cotidiana.

Asimismo, la vergüenza no solo afecta las relaciones interpersonales, sino que también socava la autoestima y la identidad de los individuos^6^. Las personas tienden a percibirse como “menos que los demás”, lo que refuerza ciclos de autoevaluación negativa. Tal como señala un entrevistado, “*me sentía como que no era nadie*” (E1), destacando cómo esta emoción actúa como una barrera para la autovaloración y la autorrealización. Este impacto emocional se agrava por la percepción de insuficiencia económica, ya que la falta de recursos para acceder a tratamientos dentales perpetúa la sensación de impotencia y resignación. La frase de otro entrevistado, “*no tenía medios para pagar algo particular y rápido*” (E8), subraya las barreras económicas que existen y no solo limitan las opciones de tratamiento, sino que también intensifican el sufrimiento psíquico^52^^,^^57^.

El aislamiento social emerge como una consecuencia directa de la vergüenza, profundizando el malestar emocional y limitando las oportunidades de conexión humana. Los relatos reflejan por qué las personas prefieren evitar situaciones donde puedan ser juzgadas, lo que lleva a una desconexión autoimpuesta que agrava la soledad. Este ciclo de exclusión social refuerza el sufrimiento psíquico, generando un impacto multidimensional en la calidad de vida. Por ejemplo, una entrevistada mencionó que prefería no asistir a reuniones sociales debido a la falta de dientes. Aquel testimonio evidencia el sentimiento de vergüenza que no solo limita la interacción social, sino que también afecta la percepción del propio valor, creando barreras emocionales difíciles de superar.

Resulta necesario comprender que la vergüenza vinculada a la pérdida dental no es una emoción aislada, sino un fenómeno que dialoga con los determinantes sociales de la salud, ya que, como diría Le Bretón, expone al individuo a una violencia relacional constante, en la que cada interacción recuerda la indignidad social asignada al cuerpo^25^. Las trayectorias de vida marcadas por la pobreza, la exclusión laboral y la falta de acceso oportuno a prestaciones preventivas terminan materializándose en cuerpos que llevan inscritas esas desigualdades. La falta de dientes, en este sentido, no solo habla de un problema odontológico, sino que constituye una huella visible de la precariedad, lo que expone a las personas a juicios sociales que refuerzan la estigmatización y la autoexclusión.

A partir de ello, se observa que la vergüenza no solo deteriora la dimensión subjetiva y emocional, sino que también se convierte en un obstáculo estructural en la atención en salud. Muchos entrevistados manifestaron que evitaban acudir al dentista precisamente por temor a ser juzgados por su condición bucal. Esta paradoja evidencia cómo la vergüenza opera como una barrera adicional al ya restringido acceso económico y territorial, reproduciendo un círculo vicioso en el que el sufrimiento psíquico y la precariedad dental se retroalimentan mutuamente. De este modo, la emoción deja de ser una experiencia interna para convertirse en un mecanismo social de control y exclusión, finalmente, en un problema de salud mental.

Por último, es posible señalar que la experiencia de la vergüenza por la pérdida dental debe ser considerada dentro de un marco de derechos. La salud oral, al estar estrechamente vinculada al bienestar psicoemocional, no puede seguir siendo tratada como un ámbito secundario o estético. Reconocer la salud bucal como parte integral de la salud general permitiría visibilizar las consecuencias emocionales y sociales que acarrea su descuido, promoviendo políticas públicas que no solo se orienten a la rehabilitación protésica, sino también a la prevención, la dignificación de la atención y la reducción del estigma asociado, en definitiva, a la mejora del bienestar y salud mental. De este modo, se avanzaría hacia una comprensión más inclusiva de la salud, en la que la vergüenza no sea una condena silenciosa, sino un punto de partida para la transformación social.

## Conclusiones

La investigación presentada permite sostener que la pérdida dental no constituye únicamente una condición clínica o estética, sino que se configura como un fenómeno profundamente social y emocional, atravesado por la vergüenza, el estigma y la desigualdad estructural. Los testimonios recogidos muestran cómo la ausencia de dientes impacta de manera directa en la autoestima, la identidad y la capacidad de participar plenamente en la vida social, transformando la salud oral en un marcador de diferenciación social y de exclusión.

En este marco, la vergüenza emerge como una emoción central que atraviesa la experiencia de las personas afectadas. Esta emoción no solo surge de la percepción del juicio externo, sino que también se internaliza, produciendo sentimientos de insuficiencia personal, tristeza, rabia y autodesvalorización. Los relatos de quienes se cubren la boca, evitan reír o se autoexcluyen de espacios comunitarios, evidencian cómo la vergüenza moldea el cuerpo y la subjetividad, generando mecanismos de autocensura y aislamiento que deterioran la calidad de vida, especialmente en las personas mayores.

El estigma vinculado a la salud bucal se expresa tanto en prácticas sociales como en instituciones de salud. La investigación muestra que las personas mayores con pérdida dental son objeto de juicios morales, asociados a pobreza, falta de higiene o descuido, que refuerzan la discriminación estructural. A nivel institucional, la insuficiencia del sistema público y los elevados costos en el sector privado reproducen este estigma, ya que quienes no pueden acceder a tratamientos deben convivir con una apariencia que los coloca en una posición de vulnerabilidad social y simbólica.

En este sentido, la vergüenza no puede comprenderse únicamente como una emoción individual, sino como el reflejo de una desigualdad estructural, especialmente en las personas mayores, que limita el acceso a la salud dental y perpetúa ciclos de exclusión. El hecho de que la dentadura se perciba socialmente como un requisito de dignidad y ciudadanía estética refuerza el carácter normativo y clasista de la salud oral, situando a quienes carecen de recursos en un espacio de invisibilización y marginación, en el cual las personas mayores se ven enfrentadas, deteriorando su salud mental.

Los hallazgos también revelan que la vergüenza en las personas mayores, tienen consecuencias psicológicas profundas, expresadas en depresión, ansiedad y sufrimiento psíquico. El aislamiento social, la imposibilidad de compartir espacios cotidianos como comidas o celebraciones, y la autocensura en conversaciones refuerzan un ciclo de dolor emocional que va más allá de la pérdida funcional de los dientes. La salud mental, por tanto, se ve directamente afectada por la ausencia de atención dental oportuna y adecuada, consolidando un vínculo estrecho entre cuerpo, emoción y exclusión social.

Finalmente, la investigación permite concluir que la problemática de la pérdida dental reflejada en personas mayores exige ser abordada desde una perspectiva integral, que trascienda lo clínico e incluya lo psicosocial. Garantizar el acceso equitativo a tratamientos dentales asequibles, no solo implica mejorar la funcionalidad masticatoria, sino también devolver dignidad, autoestima y posibilidad de integración social a las personas mayores. Solo en la medida en que se reconozca la salud oral como parte constitutiva de la salud mental y de la calidad de vida, será posible reducir el impacto de la vergüenza, el estigma y el sufrimiento que marcan la experiencia de quienes han perdido sus dientes.
